# Supplementary material for: Lifestyle Interventions to Improve Glycemic Control in Adults with Type 2 Diabetes Living in Low-and-Middle Income Countries: A Systematic Review and Meta-Analysis of Randomized Controlled Trials (RCTs)
Source: Int J Environ Res Public Health. 2021 Jun 10;18(12):6273. doi: 10.3390/ijerph18126273 (PMC8296019; doi:10.3390/ijerph18126273)
Supplement: Supplementary file 1 [file ijerph-18-06273-s001.zip › ijerph-1188539-SI.pdf]

Lifestyle interventions to improve glycemic control in adults with type 2 diabetes living in Low-and-Middle Income Countries: A Systematic Review and Meta-analysis of Randomized Controlled Trials (RCTs)

# SUPPLEMENTARY FILE

**Table S1.** Search Strategy.
**Table S2.** Lifestyle interventions' characteristics.
**Table S3.** Outcome measures reported in included trials.
**Table S4.** Quality assessment ratings, scored using the EPHPP tool.
**Figure S1:** Funnel plots for the visual assessment of publication bias.
**Table S5.** GRADE assessment of Quality of Evidence**.**
**Table S6.** PRISMA 2020 Checklist**.**

**Table S1.** Search Strategy.

| |
|---|
| **Diabetes Type 2** |
| “Type 2 diabetes mellitus” OR “Diabetes Mellitus, Noninsulin−Dependent” OR “Diabetes Mellitus, Ketosis−Resistant” OR “Diabetes Mellitus, Ketosis Resistant” OR “Ketosis−Resistant Diabetes Mellitus” OR “Diabetes Mellitus, Non−Insulin Dependent” OR “Diabetes Mellitus, Non−Insulin−Dependent” OR “Non−Insulin−Dependent Diabetes Mellitus” OR “Diabetes Mellitus, Stable” OR “Stable Diabetes Mellitus” OR “Diabetes Mellitus, Type II” OR “NIDDM” OR “Diabetes Mellitus, Noninsulin Dependent” OR “Diabetes Mellitus, Maturity−Onset” OR “Diabetes Mellitus, Maturity Onset” OR “Maturity−Onset Diabetes Mellitus” OR “Maturity Onset Diabetes Mellitus” OR “MODY” OR “Diabetes Mellitus, Slow−Onset” OR “Diabetes Mellitus, Slow Onset” OR “Slow−Onset Diabetes Mellitus” OR “Noninsulin−Dependent Diabetes Mellitus” OR “Noninsulin Dependent Diabetes Mellitus” OR “Maturity−Onset Diabetes” OR “Diabetes, Maturity−Onset” OR “Maturity Onset Diabetes” OR “Type 2 Diabetes” OR “Diabetes, Type 2” OR “Diabetes Mellitus, Adult−Onset” OR “Adult−Onset Diabetes Mellitus” OR “Diabetes Mellitus, Adult Onset” |
| **AND** |
| **Lifestyle Interventions** |
| “Exercises” OR “Physical Activity” OR “Activities, Physical” OR “Activity, Physical” OR “Physical Activities” OR “Exercise, Physical” OR “Exercises, Physical” OR “Physical Exercise” OR “Physical Exercises” OR “Acute Exercise” OR “Acute Exercises” OR “Exercise, Acute” OR “Exercises, Acute” OR “Exercise, Isometric” OR “Exercises, Isometric” OR “Isometric Exercises” OR “Isometric Exercise” OR “Exercise, Aerobic” OR “Aerobic Exercise” OR “Aerobic Exercises” OR “Exercises, Aerobic” OR “Exercise Training” OR “Exercise Trainings” OR “Training, Exercise” OR “Trainings, Exercise” OR “Cool−Down Exercise” OR “Gymnastics” OR “Muscle Stretching Exercises” OR “Physical Conditioning, Human” OR “Circuit−Based Exercise” OR “Endurance Training” OR “High−Intensity Interval Training” OR “Plyometric Exercise” OR “Resistance Training” OR “Running” OR “Jogging” OR “Swimming” OR “Walking” OR “Stair Climbing” OR “Warm−Up Exercise” |
| **AND** |
| “Diet” OR “Diet Fads” OR “Diet, Atherogenic” OR “Diet, Carbohydrate Loading” OR “Diet, High−Protein Low−Carbohydrate” OR “Diet, Ketogenic” OR “Diet, Cariogenic” OR “Diet, Diabetic” OR “Diet, Fat−Restricted” OR “Diet, Gluten−Free” OR “Diet, High−Fat” OR “Diet, High−Protein” OR “Diet, High−Protein Low−Carbohydrate” OR “Diet, Mediterranean” OR “Diet, Protein−Restricted” OR “Diet, Reducing” OR “Diet, Sodium−Restricted” OR “Diet, Vegetarian” OR “Diet, Macrobiotic” OR “Diet, Vegan” OR “Diet, Western” OR “Dietary Approaches To Stop Hypertension” OR “Energy Intake” OR “Caloric Restriction” OR “Fasting” OR “Healthy Diet” OR “Portion Size” OR “Serving Size” |
| **OR** |
| “Healthy Lifestyle” OR “Healthy Aging” OR “Healthy Diet” OR “Lifestyle, Healthy” OR “Lifestyles, Healthy” OR “Healthy Life Styles” OR “Healthy Lifestyles” OR “Healthy Life Style” OR “Life Style, Healthy” OR “Life Styles, Healthy” |
| **OR** |
| “Cessation, Smoking” OR “Smoking Cessations” OR “Stopping Smoking” OR “Smoking, Stopping” OR “Giving Up Smoking” OR “Smoking, Giving Up” OR “Smoking, Giving Up” OR “Up Smoking, Giving” OR “Quitting Smoking” OR “Smoking, Quitting” OR “Smoking Prevention” |
| **OR** |
| “Education, Health” OR “Community Health Education” OR “Education, Community Health” OR “Health Education, Community” OR “Consumer Health Information” OR “Health Literacy” OR “Health Education, Dental” OR “Health Fairs” OR “Health Promotion” OR “Healthy People Programs” OR “Weight Reduction Programs” OR “Patient Education as Topic” OR “Prenatal Education” |
| **AND** |
| **Low−and Middle Income Countries** |
| “Countries, Developing” OR “Country, Developing” OR “Developing Country” OR “Least Developed Countries” OR “Countries, Least Developed” OR “Country, Least Developed” OR “Developed Countries, Least” OR “Developed Country, Least” OR “Least Developed Country” OR “Less−Developed Countries” OR “Countries, Less−Developed” OR “Country, Less−Developed” OR “Less Developed Countries” OR “Less−Developed Country” OR “Under−Developed Nations” OR “Nation, Under−Developed” OR “Nations, Under−Developed” OR “Under Developed Nations” OR “Under−Developed Nation” OR “Third−World Countries” OR “Countries, Third−World” OR “Country, Third−World” OR “Third World Countries” OR “Third−World Country” OR “Third−World Nations” OR “Nation, Third−World” OR “Nations, Third−World” OR “Third World Nations” OR “Third−World Nation” OR “Under−Developed Countries” OR “Countries, Under−Developed” OR “Country, Under−Developed” OR “Under Developed Countries” OR “Under−Developed Country” OR “Developing Nations” OR “Developing Nation” OR “Nations, Developing” OR “Less−Developed Nations” OR “Less Developed Nations” OR “Less−Developed Nation” OR “Nation, Less−Developed” OR “Nations, Less−Developed” OR “Indigent*” OR “Indigency” OR “Low−Income Population” OR “Low−Income Populations” OR “Population, Low−Income” OR “Populations, Low−Income” OR “Low Income Population” OR “Low Income Populations” OR “Population, Low Income” OR “Populations, Low Income |

**Table S2.** Lifestyle interventions' characteristics.

| Reference (Year) | Delivered by | Location | Duration | PA | Diet | Medication | Foot/ Skin care | Glucose Monitoring | Smoking Cessation | Stress Control | Monitor BP | Other |
|---|---|---|---|---|---|---|---|---|---|---|---|---|
| Chaveepojnkamjorn (2009) | Not stated | Healthcare and hospital setting | 5 x 2−hour sessions monthly | √ | √ | | | | | | | Building good relationships, diabetes education, motivation for self−care activities |
| Chen (2019) | HCP/MDT | Healthcare and hospital setting | 1 x 2 hour lecture every 2 months, with periodical follow−up interviews and an annual physical examination | √ | √ | | | | | | | Health seeking behaviour and psychological counselling |
| Chow (2016) | HCP/ MDT | Home visits | 2 home visits over 3 months. Average session = 62.47 (5.24) minutes | | √ | √ | | | | | | Education on T2DM and complications |
| Debussche (2018) | TPs | Health care setting | 3 x 1.5 – 2−hour courses delivered over 1 year | √ | √ | √ | | √ | | | | Cardiovascular risk management |
| Essien (2017) | HCP/MDT | Hospital setting | 12 x 2−hour education sessions (different themes) every 2 weeks, over 6 months | √ | √ | √ | √ | √ | √ | | | |
| Fottrell (2019) | TPLP | 1) At home (to mobile) | 1) 2 x weekly 1minute voice messages, x 14 months | | | | | | | | | Lifestyle advice − no further details provided |
| | | 2) Community health centres | 2) 1 x 2−hour session monthly x 14 months | | | | | | | | | |
| Gagliardino (2013) | TPLP | Not stated | 4 x 90−120−minutes per week, with reinforcement session at 6 months | √ | √ | | √ | √ | | | | Advantages of weight loss and self−care and education on the effects of obesity |
| Gathu (2018) | TPLP | Hospital setting | 3 x 1−hour sessions every 6 weeks over 6 months | | √ | √ | √ | √ | √ | | | |
| Goldhaber−Fiebert (2003) | HCP/MDT | Community health centres | 11 x 90−minute nutrition classes weekly x 12 weeks +/− 3 x 60 minutes walking sessions per week x 12 weeks | √ | √ | √ | | | | | | Both intervention and control received a lecture on T2DM, it's symptoms, complications and treatment |

| | | | | | | | | | | | | |
|---|---|---|---|---|---|---|---|---|---|---|---|---|
| Goodarzi (2012) | TPLP | Anywhere where access was possible by mobile phone | 4 x weekly text messages x 12 weeks | √ | √ | √ | | | √ | | | |
| Grillo (2016) | HCP / MDT | Study centre | 2−hour meeting weekly x 5 weeks, reinforcement meetings every 4 months for 1 year | √ | √ | √ | | √ | | | | Overview of chronic diabetes complications |
| Huimin (2014) | Not stated | Not stated | 1) 50% V02 max for 45 minutes, 3−5 times per week x 12 weeks<br>2) Supervised exercise—vigorous intensity (75% VO2 max for 45mins x 3 weekly x 12−weeks | √ | | √ | | | | | | |
| Jain (2018) | TPLP | Home visits | 1 x home visit every 6 weeks, telephonic follow up and reminders every 15 days | √ | √ | √ | | | | | √ | Overview of chronic diabetes complications |
| Ju (2018) | TPLP | Community health centres | At least once monthly x 12 months | √ | √ | √ | | | √ | | | |
| Li (2016) | HCP / MDT | Study centre | Nutrition classes every morning for 30 days, followed by discussion periods | √ | √ | √ | | | √ | | | Participants lived together for 1 month where they received nutrition lectures about diabetes |
| Malathy (2011) | HCP / MDT | Hospital and clinic setting | 1 x 20−minute session per month x 3 months | √ | √ | | | √ | √ | | √ | Education on T2DM and complications |
| Mash (2014) | TPLP | Community health centres | 4 x 60−minute sessions | | | √ | | | | | | |
| Mohammadi (2018) | Not stated | Diabetes clinic | 8 x 2−hour sessions | √ | √ | √ | | | √ | | | |
| Muchiri (2015) | HCP / MDT | Health care setting | 8 x 2− hour weekly sessions, 4 x 1.5−hour monthly sessions & 2 x 1.5 hour bi−monthly sessions | | √ | | | | | | | |
| Ojieabu (2017) | HCP / MDT | Endocrinology clinics | 4 x sessions over 4 months | √ | √ | √ | | | | | | |
| Ramadas (2018) | HCP / MDT | Medical or diabetes clinics | 12 x diet plans over 6 months | | √ | | | | | | | |
| Salahshouri (2018) | HCP / MDT | Health care setting | 8 x 1−hour sessions | | √ | | | | | | | Education on self−efficacy, |

| | | | | | | | | | | | | |
|---|---|---|---|---|---|---|---|---|---|---|---|---|
| | | | | | | | | | | | | behavioural change, economic management, religious laws, family support |
| Sanaeinasab (2020) | Not stated | Healthcare and hospital setting | Group face−to−face 1−hour weekly session for 6−weeks | √ | √ | √ | √ | √ | | √ | | |
| Samtia (2013) | HCP / MDT | Diabetes clinic | Received self−management education over 5 months | √ | √ | √ | √ | √ | √ | | | Education on T2DM and complications |
| Ur Rehman (2017) | TPLP | Hospital Research Centre | 3 days per week x 25 weeks | √ | √ | √ | | | | | | |
| Wattana (2007) | HCP / MDT | Community based | 1 x 120−minute education class, 4 x 90 −minute small group discussions, and 2 x 45− minute home visits per week | √ | √ | √ | √ | | | √ | | |
| Wichit (2017) | HCP / MDT | Hospital setting | 3 x 2−hour education sessions (week 1, 5 and 9) | √ | √ | | √ | √ | | | | Coping with diabetes−related complications |
| Zhang (2018) | HCP / MDT | Hospital setting | Every month over 2 years | √ | √ | √ | √ | √ | √ | | √ | |
| Zheng (2019) | Not stated | Cardiac rehabilitation centre | 1 x education session on initial visit, 1 x 45−minute education session, 1 x 40−minute practical session, 1 x 60−minute education session | √ | √ | √ | √ | √ | | | | Diabetes prevention, treating diabetes complications and developing personalised strategies for decision making |
| Zhong (2015) | TPLP | Community health centres | A.1.5 – 2 hours biweekly sessions x 12 weeks over 6 months | √ | √ | √ | √ | | | √ | | Depression, barriers to self−management, and obtaining resources and support |

HCP / MDT: healthcare professional or a multidisciplinary team, intervention delivered by either a HCP or a MDT that included a heath care professional. TPLP: trained peer or lay person, intervention delivered by a non−HCP but by a peer or lay person who had had training in diabetes education.

**Table S3.** Outcome measures reported in included trials.

| Reference | Year | Outcome Measure(s) | Mean Pre–Value (SD) | Mean Post–Value (SD) | Change in Value | Lower 95% Confidence Interval | Upper 95% Confidence Interval | *P*–Value |
|---|---|---|---|---|---|---|---|---|
| Chaveepojnkamjorn | 2009 | Quality of Life (EQ–5D–3L) | 80.6 (7.5) | 96.2 (5.8) | – | – | – | <0.001 |
| Chen | 2019 | Fasting Blood Glucose (mmol/L) | 8.3 | 7.9 | – | – | – | 0.009 |
| Chow | 2016 | HbA1c (%) | 8.92 (2.02) | 8.19 (1.43) | – | – | – | <0.001 |
| Debussche | 2018 | BMI (kg/m²) | 28.3 (5.4) | – | −1.65 | −2.25 | −1.06 | 0.005 |
| | | Waist circumference (cm) | 93.7 (12.1) | – | −3.34 | −5.56 | −1.13 | 0.003 |
| | | Systolic Blood Pressure (mmHg) | 133 (27) | – | −6.46 | −11.63 | −1.28 | 0.003 |
| | | Diastolic Blood Pressure (mmHg) | 82 (11) | – | −0.4 | −2.27 | 3.07 | 0.36 |
| | | HbA1c (%) | 10.6 (1.8) | – | −1.05 | −1.54 | −0.54 | 0.006 |
| Essien | 2017 | HbA1c (%) | 10.9 (1.7) | 8.4 | – | 8.1 | 8.9 | <0.0001 |
| Fottrell | 2019 | BMI (kg/m²) | 21.9 (3.6) | – | −0.05 | −0.28 | 0.17 | 0.631 |
| | | Systolic Blood Pressure (mmHg) | 124.4 (20) | – | −1.05 | −3.46 | 1.35 | 0.391 |
| | | Diastolic Blood Pressure (mmHg) | 72.9 (11.2) | – | −1.12 | −2.59 | 0.35 | 0.135 |
| | | Quality of Life (EQ–5D) | 0.85 | – | −0.01 | −0.06 | 0.04 | 0.687 |
| | | BMI (kg/m²) | 22 (3.6) | – | 0.08 | −0.21 | 0.38 | 0.572 |
| | | Systolic Blood Pressure (mmHg) | 125.3 (19.6) | – | −0.19 | −2.54 | 2.17 | 0.877 |
| | | Diastolic Blood Pressure (mmHg) | 73.8 (11.2) | – | −0.25 | −1.78 | 1.28 | 0.748 |
| | | Quality of Life (EQ–5D) | 0.8 | – | 0.03 | −0.02 | 0.08 | 0.253 |
| Gagliardino | 2013 | HbA1c (%) | 7.8(1.4) | – | −0.34 | – | | – |
| | | Systolic Blood Pressure (mmHg) | 145(19) | – | −9 | – | – | – |
| | | Triglycerides (mmol/L) | 1.959 (0.875) | – | −0.03387 | – | – | <0.05 |
| | | HbA1c (%) | 7.5(1.5) | – | −0.68 | – | – | – |
| | | Systolic Blood Pressure (mmHg) | 142(14) | – | −2 | – | – | – |
| | | Triglycerides (mmol/L) | 1.9204 (0.4584) | – | −0.13548 | – | – | <0.05 |
| | | HbA1c (%) | 7.7(1.3) | – | −0.84 | – | – | 0.331 |

| | | | | | | | | |
|---|---|---|---|---|---|---|---|---|
| | | Systolic Blood Pressure (mmHg) | 141(18) | – | −16 | – | – | <0.05 |
| | | Triglycerides (mmol/L) | 1.898 (0.5363) | – | −0.2032 | – | – | <0.05 |
| Gathu | 2018 | BMI (kg/m²) | 28.5 (3.73) | 28.9 (3.87) | – | – | – | 0.86 |
| | | Systolic Blood Pressure (mmHg) | 134.3 (14.63) | 132.6 (15.320 | – | – | – | 0.57 |
| | | Diastolic Blood Pressure (mmHg) | 80.7 (10.53) | 78 (9.04) | – | – | – | 0.39 |
| | | HbA1c (%) | 9.7 (1.78) | 8.8 (1.89) | – | – | – | 0.37 |
| Goldhaber−Fiebert | 2003 | Body Weight (kg) | 72.4 (14.6) | – | −1 (2.2) | −2.48 | −0.15 | 0.028 |
| | | BMI (kg/m²) | 30.1 (5.1) | – | −0.4 (0.9) | −1.06 | −0.08 | 0.022 |
| | | Systolic Blood Pressure (mmHg) | 138 (19) | – | −5 (23) | −10.75 | 10.06 | 0.95 |
| | | Diastolic Blood Pressure (mmHg) | 84 (10) | – | −7 (9) | −8.57 | 0.24 | 0.06 |
| | | Total cholesterol (mmol/L) | 6.837 (1.3447) | – | 0.2069 (0.931) | 0.6995 | 0.2242 | 0.31 |
| | | HDL cholesterol (mmol/L) | 45 (12) | – | −5 (5) | −4.05 | 1.95 | 0.49 |
| | | LDL cholesterol (mmol/L) | 4.024 (1.2154) | – | 5 (36) | −12.43 | 24.02 | 0.53 |
| | | Triglycerides (mmol/L) | 3.25 (2.76) | – | −0.54 (1.84) | −1.83 | 0.13 | 0.09 |
| | | HbA1c (%) | 8.6 (3.7) | – | −1.8 (2.3) | −2.52 | −0.15 | 0.028 |
| Goodarzi | 2012 | Total cholesterol (mmol/L) | 4.68 (1.07) | 4.29 (0.9873) | – | – | – | 0.002 |
| | | HDL cholesterol (mmol/L) | 1.13 (0.28) | 1.13 (0.24) | – | – | – | 0.448 |
| | | LDL cholesterol (mmol/L) | 2.531 (0.8342) | 2.274 (0.7745) | – | – | – | 0.19 |
| | | Triglycerides (mmol/L) | 2.03 (1.13) | 1.8082 (0.8182) | – | – | – | 0.372 |
| | | HbA1c (%) | 7.91 (1.24) | 7.02 (1.02) | – | – | – | 0.24 |
| Grillo | 2016 | BMI (kg/m²) | 30.7(5.7) | 30.4(6.3) | – | – | – | <0.01 |
| | | Waist circumference (cm) | 105(13) | 103.9(13.6) | – | – | – | 913 |
| | | Systolic Blood Pressure (mmHg) | 141(19) | 135(18) | – | – | – | <0.001 |
| | | Diastolic Blood Pressure (mmHg) | 81(12) | 79(11) | – | – | – | <0.001 |
| | | HDL cholesterol (mmol/L) | 43(12) | 43(12) | – | – | – | <0.001 |
| | | LDL cholesterol (mmol/L) | 108(37) | 108(40) | – | – | – | <0.001 |
| Huimin | 2014 | Body Weight (kg) | 78.8 (2.4) | 79.7 (2.4) | – | – | – | – |
| | | BMI (kg/m²) | 27.2 (0.7) | 27.4 (0.7) | – | – | – | – |
| | | Waist circumference (cm) | 95.8 (2.2) | 94.2 (1.9) | – | – | – | – |
| | | Systolic Blood Pressure (mmHg) | 131 (3) | 129 (4) | – | – | – | <0.05 |

| | | | | | | | | |
|---|---|---|---|---|---|---|---|---|
| | | Diastolic Blood Pressure (mmHg) | 80 (1) | 77 (2) | – | – | – | <0.05 |
| | | Fasting Blood Glucose (mmol/L) | 10.3 (0.9) | 9.6 )0.7) | – | – | – | – |
| | | HbA1c (%) | 8.8 (0.5) | 7.7 (0.4) | – | – | – | – |
| | | V02max (ml/kg/min) | 23.1 (1) | 25 (1.3) | – | – | – | – |
| Jain | 2018 | Body Weight (kg) | 59.29 (16.3) | 58.68 (13.68) | – | – | – | 0.09 |
| | | BMI (kg/m²) | 23.91 (4.48) | 23.84 (4.5) | – | – | – | 0.11 |
| | | Waist circumference (cm) | 84.71 (12.39) | 85.72 (13.86) | – | – | – | 0.9 |
| | | Systolic Blood Pressure (mmHg) | 125.14 (16.8) | 126.04 (22.79) | – | – | – | 0.63 |
| | | Diastolic Blood Pressure (mmHg) | 77.04 (9.99) | 80.23 (39.24) | – | – | – | 0.53 |
| | | Total cholesterol (mmol/L) | 4.753 (1.259) | 4.477 (1.246) | – | – | – | 0.02 |
| | | HDL cholesterol (mmol/L) | 1.07 (0.35764) | 1.2255 (0.7316) | – | – | – | 0.03 |
| | | LDL cholesterol (mmol/L) | 2.772 (1.1065) | 2.544 (0.8384) | – | – | – | 0.01 |
| | | Triglycerides (mmol/L) | 1.897 (1.2802) | 1.6355 (1.1508) | – | – | – | 0.0007 |
| | | Fasting Blood Glucose (mmol/L) | 9.83127 (4.08369) | 8.2323 (3.724) | – | – | – | 0 |
| | | HbA1c (%) | 8.29 (2.42) | 7.63 (2.16) | – | – | – | 0 |
| Ju | 2018 | Fasting Blood Glucose (FBG) (mmol/L) | 7.89 (2.1) | 7 (2.3) | – | – | – | 0.008 |
| | | 2 hour Fasting Blood Glucose (mmol/L) | 12 (4.5) | 10 (3.9) | – | – | – | 0.252 |
| | | HbA1c (%) | 6.8 (1.5) | 6.6 (1.2) | – | – | – | 0.868 |
| Li | 2016 | Body Weight (kg) | 69.1(11.5) | 68.3(10.6) | −0.8(2.9) | – | – | 0.012 |
| | | BMI (kg/m²) | 25.7(3.7) | 25.3(3.4) | −0.3(1.1) | – | – | 0.02 |
| | | Fasting Blood Glucose (FBG) (mmol/L) | 9.4(3.1) | 8.3(2.8) | −1.1(2.4) | – | – | <0.001 |
| | | 2 hour Fasting Blood Glucose (mmol/L) | 17.9(5.6) | 15.6(4.7) | −2.3(3.9) | – | – | <0.001 |
| | | HbA1c (%) | 7.9(0.4) | 7.3(0.9) | −0.6(0.9) | – | – | <0.001 |
| | | Triglyceride (mmol/L) | 1.8(0.9) | 1.6(0.9) | −0.2(0.9) | – | – | 0.008 |
| | | HDL cholesterol (mmol/L) | 1.3(0.2) | 1.3(0.3) | 0.1(0.1) | – | – | <0.001 |
| | | LDL cholesterol (mmol/L) | 3.0(0.7) | 3.0(0.8) | 0(0.7) | – | – | 0.935 |
| Malathy | 2011 | Triglyceride (mmol/L) | 1.7037 | 1.5874 | – | – | – | <0.01 |
| | | HDL cholesterol (mmol/L) | 0.9025 | 0.9465 | – | – | – | <0.05 |
| | | LDL cholesterol (mg/dl) | 3.649 | 3.1265 | – | – | – | <0.001 |
| | | Total cholesterol (mmol/L) | 5.332 | 4.802 | – | – | – | <0.001 |
| Mash | 2014 | Waist Circumference (cm) | 101.9(14.1) | 103.6(15.5) | – | – | – | 0.396 |

| | | | | | | | |
|---|---|---|---|---|---|---|---|
| | | Body weight (kg) | 84.4(18.7) | 83.8(20.2) | − | − | − | 0.392 |
| | | Systolic Blood Pressure (mmHg) | 140.2(22.4) | 143.1(24.2) | − | − | − | 0.044 |
| | | Diastolic Blood Pressure (mmHg) | 85.9(11.7) | 85(11.9) | − | − | − | 0.002 |
| | | Total Cholesterol (mmol/L) | 5(1.1) | 4.8(1.1) | − | − | − | 0.066 |
| | | HBA1c (%) | 8.9(2.3) | 8.4(2) | − | − | − | 0.967 |
| Mohammadi | 2018 | Body weight (kg) | 72.2(7.84) | 68.3(6.81) | − | − | − | 0.001 |
| | | BMI (kg/m2) | 27.1(1.92) | 25.7(1.77) | − | − | − | 0.001 |
| | | Waist Circumference (cm) | 101(6.10) | 98.7(5.71) | − | − | − | 0.001 |
| | | LDL Cholesterol (mmol/L) | 7.4(0.54) | 5.6(0.42) | − | − | − | 0.001 |
| | | HDL Cholesterol (mmol/L) | 1.2(0.15) | 1.3(0.16) | − | − | − | 0.001 |
| | | Fasting Blood Glucose (mmol/L) | 9.66(1.88) | 7.6(0.68) | − | − | − | 0.001 |
| | | HBA1c (%) | 7.97(1.01) | 7.29(0.66) | − | − | − | 0.001 |
| | | Quality of Life (DQOL Total) | 52.8(3.42) | 57.6(3.99) | − | − | − | 0.001 |
| Muchiri | 2015 | HBA1c (%) | 10.8(1.8) | 9.8(0.3) | − | − | − | 0.16 |
| | | BMI (kg/m2) | 31.5(7) | 30.6(0.3) | − | − | − | 0.18 |
| | | Total Cholesterol (mmol/L) | 4.8(1.2) | 4.74(0.1) | − | − | − | 0.37 |
| | | LDL Cholesterol (mmol/L) | 3(0.90) | 2.74(0.08) | − | − | − | 0.49 |
| | | HDL Cholesterol (mmol/L) | 1.1(0.27) | 1.02(0.03) | − | − | − | 0.17 |
| | | Systolic Blood Pressure (mmHg) | 142.9(22.9) | 141.1(2.9) | − | − | − | 0.89 |
| Ojieabu | 2017 | BMI (kg/m2) | 27.1(4.3) | 26.7(5.6) | − | − | − | 0.624 |
| | | Systolic Blood Pressure (mmHg) | 146.4(13.9) | 133.8(18.5) | − | − | − | <0.001 |
| | | Diastolic Blood Pressure (mmHg) | 85.8(13.3) | 79.8(9.4) | − | − | − | 0.002 |
| | | Fasting Blood Glucose (mmol/L) | 8.7(1.69) | 7.32(2.23) | − | − | − | <0.001 |
| Ramadas | 2018 | HBA1c (%) | 9.1(2) | 8.5(1.8) | − | − | − | 0.004 |
| | | Fasting Blood Glucose (mmol/L) | 8.9(3.9) | 7.9(2.5) | − | − | − | 0.015 |
| Salahshouri | 2018 | HBA1c (%) | 7.15(1.68) | 6.18(0.9) | − | − | − | 0 |
| | | Fasting Blood Glucose (mmol/L) | 8.21(2.8) | 6.27(1.34) | − | − | − | 0 |
| Samtia | 2013 | BMI (kg/m²) | 31.98 (7.85) | 29.25 (6.78) | −2.46 | − | − | 0.002 |
| | | Waist circumference (cm) | 95.5548 (16.002) | 89.8652 (94.0054) | −5.6896 | − | − | <0.001 |
| | | Fasting Blood Glucose (mmol/L) | 9.9206 (3.3798) | 8.8516 (3.0068) | −19.26 | − | − | 0.003 |

| | | | | | | | | |
|---|---|---|---|---|---|---|---|---|
| | | HbA1c (%) | 8.51 (1.62) | 7.5 (1.26) | −1.01 | – | – | <0.001 |
| Sanaeinasab | 2020 | HbA1c (%) | 8.8 (1.6) | 7.9 (1.4) | | 0.73 | 0.93 | <0.0001 |
| | | Fasting Blood Glucose (mmol/L) | 11.9 (3.2) | 9.6 (2.5) | | 1.7 | 2.8 | <0.0001 |
| | | HDL Cholesterol (mmol/L) | 1.3 (0.4) | 1.2 (0.3) | | −0.3 | −0.2 | <0.0001 |
| | | LDL Cholesterol (mmol/L) | 3.5 (0.8) | 3.3 (0.7) | | 0.2 | −0.3 | 0.004 |
| | | BMI (kg/m²) | 29.2 92.8) | 28.3 (2.6) | | 0.45 | −1/17 | 0.001 |
| Ur Rehman | 2017 | BMI (kg/m²) | 29.95 | 27.73 | – | – | – | 0.017 |
| Wattana | 2007 | HbA1c (%) | 8.08 (1.87) | 7.40 (1.25) | −0.68 | – | – | 0.014 |
| Wichit | 2017 | HbA1c (%) | 7.0 (2.0) | 7.0 (1.2) | – | – | – | 0.2 |
| | | Quality of life (SF−12) (PCS) | 46.7 (6.6) | 49.9 (6.9) | – | – | – | 0.04 |
| | | Quality of life (SF−12) (MCS) | 54.1 (8.6) | 58.4 (7.2) | – | – | – | 0.03 |
| Zhang | 2018 | HbA1c (%) | 7.86 (1.2) | 6.91 (0.7) | −0.95 (0.4) | – | – | 0.03 |
| | | Total cholesterol (mmol/L) | 11.8 (3.6) | 11.4 (3.8) | −0.4 (1.5) | – | – | 0.77 |
| | | LDL cholesterol (mmol/L) | 7.2 (0.5) | 6.3 (0.8) | −0.8 (0.3) | – | – | <0.01 |
| | | HDL cholesterol (mmol/L) | 3.5 (0.9) | 3.6 (0.7) | 0.1 (0.3) | – | – | 0.77 |
| | | Systolic Blood Pressure (mmHg) | 137.5 (15.4) | 125.0 (8.26) | −12.5 (5.1) | – | – | 0.02 |
| | | Diastolic Blood Pressure (mmHg) | 89.0 (14.9) | 85.8 (12.4) | −3.25 (5.6) | – | – | 0.57 |
| | | BMI (kg/m²) | 25.33 (4.5) | 24.67 (4.3) | −0.67 (1.8) | – | – | 0.71 |
| Zheng | 2019 | Fasting Blood Glucose (mmol/L) | 8.35 (1.21) | 6.11 (0.72) | – | – | – | <0.01 |
| | | 2HBG (mmol/L) | 13.67 (1.12) | 9.04 (1.40) | – | – | – | <0.01 |
| | | HbA1C (%) | 8.30 (1.02) | 6.34 (0.87) | – | – | – | <0.01 |
| Zhong | 2015 | BMI (kg/m²) | 24.3 (2.88) | 23.7 (2.80) | – | – | – | 0.64 |
| | | Systolic Blood Pressure (mmHg) | 136 (14.91) | 128 (11.29) | – | – | – | <0.001 |
| | | Diastolic Blood Pressure (mmHg) | 82.5 (8.72) | 79.1 (7.33) | – | – | – | <0.001 |
| | | Fasting Blood Glucose (FBG) (mmol/L) | 7.68 (2.13) | 6.76 (1.80) | – | – | – | 0.02 |
| | | 2HBG (mmol/L) | 11.8 (3.54) | 10.7 (3.65) | – | – | – | 0.02 |

**Table S4.** Quality assessment ratings, scored using the EPHPP tool.

| Study | Year | Selection Bias | Study Design | Confounders | Blinding | Data Collection Method | Withdrawals and Dropouts | Global Ratings |
|---|---|---|---|---|---|---|---|---|
| Chaveepojnkamjorn | 2009 | Moderate | Strong | Strong | Weak | Weak | Strong | Weak |
| Chen | 2019 | Moderate | Strong | Weak | Weak | Moderate | Strong | Weak |
| Chow | 2016 | Moderate | Strong | Strong | Weak | Strong | Moderate | Moderate |
| Debussche | 2018 | Moderate | Strong | Strong | Weak | Strong | Strong | Moderate |
| Essien | 2017 | Moderate | Strong | Strong | Moderate | Strong | Strong | Strong |
| Fottrell | 2019 | Strong | Strong | Strong | Moderate | Strong | Strong | Strong |
| Gagliardino | 2013 | Moderate | Strong | Strong | Weak | Strong | Moderate | Moderate |
| Gathu | 2018 | Moderate | Strong | Strong | Moderate | Strong | Moderate | Strong |
| Goldhaber−Fiebert | 2003 | Moderate | Strong | Strong | Weak | Strong | Strong | Moderate |
| Goodarzi | 2012 | Moderate | Strong | Strong | Weak | Strong | Strong | Moderate |
| Grillo | 2016 | Moderate | Strong | Strong | Weak | Strong | Strong | Moderate |
| Huimin | 2014 | Moderate | Strong | Strong | Weak | Strong | Weak | Weak |
| Jain | 2018 | Moderate | Strong | Strong | Weak | Strong | Strong | Moderate |
| Ju | 2018 | Weak | Strong | Strong | Weak | Strong | Moderate | Weak |
| Li | 2016 | Weak | Strong | Strong | Weak | Strong | Strong | Weak |
| Malathy | 2011 | Moderate | Strong | Weak | Weak | Strong | Weak | Weak |
| Mash | 2014 | Moderate | Strong | Weak | Weak | Weak | Weak | Weak |
| Mohammadi | 2018 | Moderate | Strong | Strong | Weak | Strong | Weak | Weak |
| Muchiri | 2015 | Moderate | Strong | Strong | Weak | Strong | Strong | Moderate |
| Ojieabu | 2017 | Moderate | Strong | Strong | Weak | Strong | Strong | Moderate |
| Ramadas | 2018 | Moderate | Strong | Strong | Weak | Strong | Strong | Moderate |
| Salahshouri | 2018 | Moderate | Strong | Strong | Weak | Strong | Strong | Moderate |
| Sanaeinasab | 2020 | Moderate | Strong | Strong | Moderate | Strong | Moderate | Strong |
| Samtia | 2013 | Moderate | Strong | Strong | Weak | Strong | Strong | Moderate |
| Ur Rehman | 2017 | Moderate | Strong | Weak | Weak | Weak | Strong | Weak |
| Wattana | 2007 | Moderate | Strong | Strong | Weak | Strong | Weak | Weak |
| Wichit | 2017 | Weak | Strong | Strong | Weak | Strong | Strong | Moderate |
| Zhang | 2018 | Weak | Strong | Strong | Weak | Strong | Weak | Weak |
| Zheng | 2019 | Moderate | Strong | Strong | Weak | Strong | Strong | Moderate |
| Zhong | 2015 | Weak | Strong | Weak | Weak | Strong | Weak | Weak |

**HbA1c**

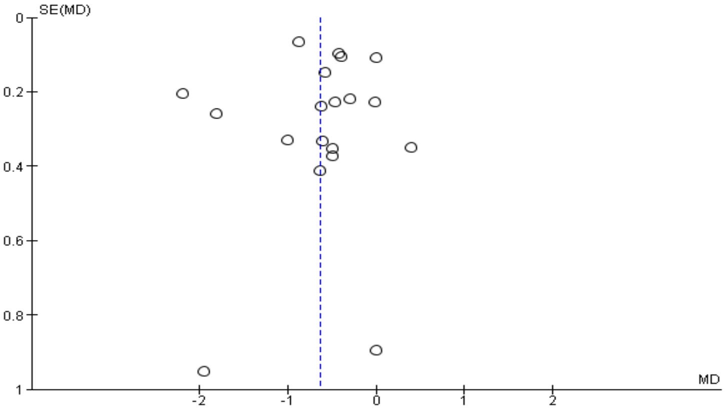


**BMI**

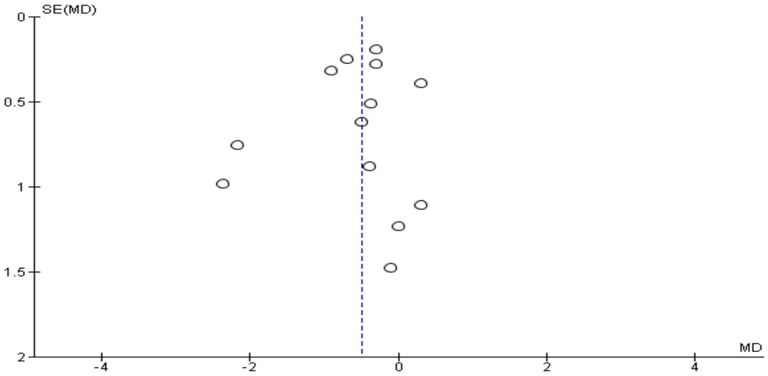


**FBG**

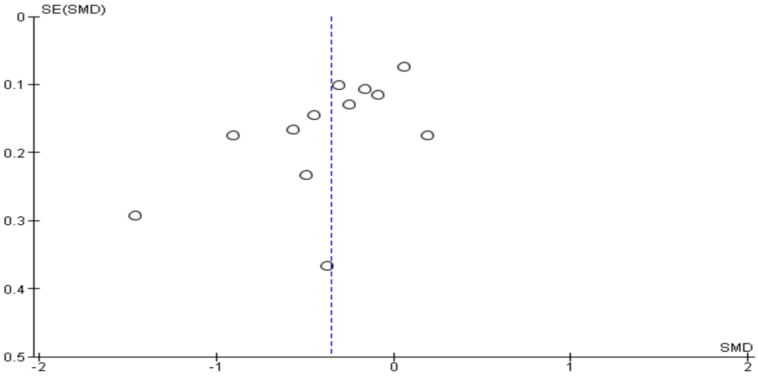


**Figure S1:** Funnel plots for the visual assessment of publication bias *.* Roughly symmetrical funnel plots indicated a low risk of publication bias and asymmetrical plots a high risk.

**Table S5.** GRADE assessment of Quality of Evidence.

| Outcome Comparisons | Studies ($n$) | MD [95% Cis] | Bias | Inconsistency | Indirectness | Imprecision | Publication Bias | Rating |
|---|---|---|---|---|---|---|---|---|
| **Overall Intervention Group** | | | | | | | | |
| Glycated Haemoglobin | 19 | −0.63 (−0.86 to −0.40) | 1 | −1[b] | 1 | 1 | 1 | 4: High |
| Body Mass Index | 13 | −0.50 (−0.80 to −0.20) | −1[a] | 1 | 1 | 1 | 1 | 4: High |
| Fasting Blood Glucose | 13 | −0.35 (−0.54 to −0.16) | −1[a] | −1[b] | 1 | 1 | 1 | 3: Moderate |
| **Intervention Type** | | | | | | | | |
| *– Self–management Education* | | | | | | | | |
| Glycated Haemoglobin | 15 | −0.69 (−0.96 to −0.43) | 1 | −1[b] | 1 | 1 | 1 | 4: High |
| Body Mass Index | 10 | −0.54 (−0.79 to −0.29) | −1[a] | 1 | 1 | 1 | 1 | 4: High |
| Fasting Blood Glucose | 10 | −0.42 (−0.69 to −0.16) | −1[a] | −1[b] | 1 | 1 | 1 | 3: Moderate |
| *– Structured diet/exercise/combined* | | | | | | | | |
| Glycated Haemoglobin | 4 | −0.07 (−0.81 to 0.67) | −1[a] | −1[b] | 1 | −1[d] | 0[e] | 1: Very Low |
| Body Mass Index | 3 | −0.06 (−0.64 to 0.51) | −1[a] | 1 | 1 | −1[d] | 0[e] | 2: Low |
| Fasting Blood Glucose | 3 | −0.14 (−0.76 to 0.48) | −1[a] | −1[b] | 1 | −1[d] | 0[e] | 1: Very Low |
| **Intervention Delivery** | | | | | | | | |
| *– Healthcare Professional(s) /MDT* | | | | | | | | |
| Glycated Haemoglobin | 9 | −0.71 (−1.01 to −0.41) | 1 | −1[b] | 1 | 1 | 0[e] | 3: Moderate |
| Body Mass Index | 6 | −0.48 (−1.11 to 0.15) | 1 | −1[b] | 1 | −1[d] | 0[e] | 2: Low |
| Fasting Blood Glucose | 7 | −0.37 (−0.70 to −0.05) | 1 | −1[b] | 1 | 1 | 0[e] | 3: Moderate |
| *– Trained Peers / lay people* | | | | | | | | |
| Glycated Haemoglobin | 6 | −0.24 (−0.47 to 0.00) | −1[a] | −1[b] | 1 | 1 | 0[e] | 2: Low |
| Body Mass Index | 3 | −0.51 (−1.10 to 0.09) | 1 | 1 | 1 | −1[d] | 0[e] | 3: Moderate |
| Fasting Blood Glucose | 2 | −0.11 (−0.33 to 0.12) | 1 | −1[b] | 1 | −1[d] | 0[e] | 3: Moderate |

| | | | | | | | | |
|---|---|---|---|---|---|---|---|---|
| **Intervention Setting** | | | | | | | | |
| *− Hospital / Clinic* | | | | | | | | |
| Glycated Haemoglobin | 10 | −0.77 (−1.08 to −0.56) | 1 | −1[b] | 1 | 1 | 0[e] | 3: Moderate |
| Body Mass Index | 7 | −0.61 (−1.07 to −0.15) | 1 | −1[b] | 1 | −1[d] | 0[e] | 2: Low |
| Fasting Blood Glucose | 7 | −0.47 (−0.74 to −0.20) | −1[a] | −1[b] | 1 | 1 | 0[e] | 2: Low |
| *− Community* | | | | | | | | |
| Glycated Haemoglobin | 3 | −0.20 (−0.59 to 0.19) | 1 | −1[b] | 1 | 1 | 0[e] | 3: Moderate |
| Fasting Blood Glucose | 2 | −0.12 (−0.48 to 0.24) | 1 | −1[b] | 1 | 1 | 0[e] | 3: Moderate |
| *− Home* | | | | | | | | |
| Glycated Haemoglobin | 3 | −0.48 (−1.44 to 0.49) | 1 | −1[b] | 1 | −1[d] | 0[e] | 2: Low |

1 = not downgraded. −1[a] = downgraded one place due to ≥ 40% of trials rated as having a high ROB on quality assessment. −1[b] = downgraded one place due to unexplained heterogeneity (>50%) −1[c] = downgraded one place due to indirectness in intervention and/or outcome. −1[d] = downgraded one place due to wide confidence interval (>0.8). 0[e] = Funnel plots not completed due to < 10 studies included in the meta−analysis.

**Table S6.** PRISMA 2020 checklist.

| Section and Topic | Item # | Checklist Item | Location Where Item is Reported |
|---|---|---|---|
| **Title** | | | |
| Title | 1 | Identify the report as a systematic review. | Page 1 |
| **Abstract** | | | |
| Abstract | 2 | See the PRISMA 2020 for abstracts checklist. | Page 1 |
| **Introduction** | | | |
| Rationale | 3 | Describe the rationale for the review in the context of existing knowledge. | Pages 1,2 |
| Objectives | 4 | Provide an explicit statement of the objective(s) or question(s) the review addresses. | Page 2 |
| **Methods** | | | |
| Eligibility criteria | 5 | Specify the inclusion and exclusion criteria for the review and how studies were grouped for the syntheses. | Page 3 |
| Information sources | 6 | Specify all databases, registers, websites, organisations, reference lists and other sources searched or consulted to identify studies. Specify the date when each source was last searched or consulted. | Pages 2,3 |
| Search strategy | 7 | Present the full search strategies for all databases, registers and websites, including any filters and limits used. | Suppl. File |
| Selection process | 8 | Specify the methods used to decide whether a study met the inclusion criteria of the review, including how many reviewers screened each record and each report retrieved, whether they worked independently, and if applicable, details of automation tools used in the process. | Page 3 |
| Data collection process | 9 | Specify the methods used to collect data from reports, including how many reviewers collected data from each report, whether they worked independently, any processes for obtaining or confirming data from study investigators, and if applicable, details of automation tools used in the process. | Pages 3,4 |
| Data items | 10a | List and define all outcomes for which data were sought. Specify whether all results that were compatible with each outcome domain in each study were sought (e.g., for all measures, time points, analyses), and if not, the methods used to decide which results to collect. | Page 3, Table 1 |
| | 10b | List and define all other variables for which data were sought (e.g., participant and intervention characteristics, funding sources). Describe any assumptions made about any missing or unclear information. | Page 3, Table 1 |
| Study risk of bias assessment | 11 | Specify the methods used to assess risk of bias in the included studies, including details of the tool(s) used, how many reviewers assessed each study and whether they worked independently, and if applicable, details of automation tools used in the process. | Pages 3, 14 Suppl. File |
| Effect measures | 12 | Specify for each outcome the effect measure(s) (e.g., risk ratio, mean difference) used in the synthesis or presentation of results. | Pages 10,11,12 |
| Synthesis methods | 13a | Describe the processes used to decide which studies were eligible for each synthesis (e.g., tabulating the study intervention characteristics and comparing against the planned groups for each synthesis (item #5)). | Pages 3,4 |
| | 13b | Describe any methods required to prepare the data for presentation or synthesis, such as handling of missing summary statistics, or data | Pages 3,4 |

| Section and Topic | Item # | Checklist Item | Location Where Item is Reported |
|---|---|---|---|
| | | conversions. | |
| | 13c | Describe any methods used to tabulate or visually display results of individual studies and syntheses. | Pages 3,4 |
| | 13d | Describe any methods used to synthesize results and provide a rationale for the choice(s). If meta−analysis was performed, describe the model(s), method(s) to identify the presence and extent of statistical heterogeneity, and software package(s) used. | Pages 3,4 |
| | 13e | Describe any methods used to explore possible causes of heterogeneity among study results (e.g., subgroup analysis, meta−regression). | Page 4 |
| | 13f | Describe any sensitivity analyses conducted to assess robustness of the synthesized results. | Page 4 |
| Reporting bias assessment | 14 | Describe any methods used to assess risk of bias due to missing results in a synthesis (arising from reporting biases). | Pages 4,14, Suppl. File |
| Certainty assessment | 15 | Describe any methods used to assess certainty (or confidence) in the body of evidence for an outcome. | Pages 4,14, Suppl. File |
| **Results** | | | |
| Study selection | 16a | Describe the results of the search and selection process, from the number of records identified in the search to the number of studies included in the review, ideally using a flow diagram. | Pages 4,5 |
| | 16b | Cite studies that might appear to meet the inclusion criteria, but which were excluded, and explain why they were excluded. | Pages 4,5 |
| Study characteristics | 17 | Cite each included study and present its characteristics. | Pages 6,7,8,9,10 |
| Risk of bias in studies | 18 | Present assessments of risk of bias for each included study. | Page 14, Suppl. File |
| Results of individual studies | 19 | For all outcomes, present, for each study: (a) summary statistics for each group (where appropriate) and (b) an effect estimate, and its precision (e.g., confidence/credible interval), ideally using structured tables or plots. | Page 12 |
| Results of syntheses | 20a | For each synthesis, briefly summarise the characteristics and risk of bias among contributing studies. | Pages 11,12,13 |
| | 20b | Present results of all statistical syntheses conducted. If meta−analysis was done, present for each the summary estimate and its precision (e.g., confidence/credible interval) and measures of statistical heterogeneity. If comparing groups, describe the direction of the effect. | Pages 12,13 |
| | 20c | Present results of all investigations of possible causes of heterogeneity among study results. | Pages 12,13 |
| | 20d | Present results of all sensitivity analyses conducted to assess the robustness of the synthesized results. | Page 14 |
| Reporting biases | 21 | Present assessments of risk of bias due to missing results (arising from reporting biases) for each synthesis assessed. | Page 14 |
| Certainty of | 22 | Present assessments of certainty (or confidence) in the body of evidence for each outcome assessed. | Page 14 |

| Section and Topic | Item # | Checklist Item | Location Where Item is Reported |
|---|---|---|---|
| evidence | | | |
| **Discussion** | | | |
| Discussion | 23a | Provide a general interpretation of the results in the context of other evidence. | Pages 14,15 |
| | 23b | Discuss any limitations of the evidence included in the review. | Pages 15,16 |
| | 23c | Discuss any limitations of the review processes used. | Page 16 |
| | 23d | Discuss implications of the results for practice, policy, and future research. | Page 16 |
| **Other Information** | | | |
| Registration and protocol | 24a | Provide registration information for the review, including register name and registration number, or state that the review was not registered. | Page 2 |
| | 24b | Indicate where the review protocol can be accessed, or state that a protocol was not prepared. | PROSPERO 2020: CRD42020151938 |
| | 24c | Describe and explain any amendments to information provided at registration or in the protocol. | N/A |
| Support | 25 | Describe sources of financial or non–financial support for the review, and the role of the funders or sponsors in the review. | N/A |
| Competing interests | 26 | Declare any competing interests of review authors. | N/A |
| Availability of data, code and other materials | 27 | Report which of the following are publicly available and where they can be found – template data collection forms; data extracted from included studies; data used for all analyses; analytic code; any other materials used in the review. | N/A |
